# Marfan Syndrome versus Loeys–Dietz Syndrome after Aortic Surgery: A Systematic Review and Meta-analysis of Reconstructed Time-to-Event Data

**DOI:** 10.1055/a-2965-6400

**Published:** 2026-10-08

**Authors:** Johannes Fischer, Steve Wehner, Ayodeji Arobo, Abdullah Almehandi, Felipe S. Passos, Christian Eitel, Hristo Kirov, Xander Jacquemyn, Ibrahim Sultan, Torsten Doenst, Tulio Caldonazo

**Affiliations:** 1 Department of Cardiothoracic Surgery 39065 Friedrich-Schiller-University JenaJenaGermany; 2 Medical School 54715 College of Health Sciences, Obafemi Awolowo UniversityIle-ifeNigeria; 3 Department of Cardiovascular Science 4919 University College LondonLondonUnited Kingdom; 4 Department of Thoracic SurgeryMaterDei HospitalSalvadorBahiaBrazil; 5 Department of Cardiology and Vascular Medicine 39081 West German Heart and Vascular Center, University Hospital EssenEssenGermany; 6 Department of Cardiothoracic Surgery 196239 UPMC Heart and Vascular Institute, University of Pittsburgh Medical CenterPittsburghPennsylvaniaUnited States; 7 Department of Cardiothoracic Surgery 39065 Jena University Hospital, Universitatsklinikum JenaJenaGermany

**Keywords:** marfan syndrome, Loeys–Dietz syndrome, aortic surgery

## Abstract

**Objective:**

Marfan syndrome (MFS) and Loeys–Dietz syndrome (LDS) are hereditary connective tissue diseases, both characterized by aortic root and vessel involvement with an increased risk of aneurysms and dissections. Despite advances in surgical techniques and improved postoperative follow-up, the impact of surgery on long-term outcomes in these patient groups is still unknown.

**Methods:**

We performed a systematic review and a meta-analysis to compare the postoperative long-term events. The main outcome was long-term survival. Additional outcomes were freedom from dissection and reoperation. After reconstruction of time-to-event data for the individual survival analysis, pooled Kaplan–Meier curves for the endpoints were generated.

**Results:**

A total of 1,794 studies were identified, of which 5 were included in the analysis, encompassing 1,005 patients (MFS: 778; LDS: 227). With MFS as the reference group, there was no significant difference in long-term survival between both groups, based on age (hazard ratio [HR]: 0.919; 95% confidence interval [CI]: 0.58–1.45; *p* =0.717) or postoperative follow-up (HR: 0.997; 95% CI: 0.45–2.19; *p* =0.995). However, LDS was associated with higher risk of dissection (HR: 0.331; 95% CI: 0.21–0.53; *p* <0.005) and reoperation (HR: 0.603; 95% CI: 0.40–0.90; *p* =0.014), reflecting unadjusted comparisons reconstructed from Kaplan–Meier curves.

**Conclusion:**

Although long-term survival is similar between MFS and LDS patients, those with LDS face a significantly higher risk of aortic dissection and reoperation. This elevated risk may be partly explained by the intrinsic genotypic heterogeneity of the LDS population. However, our pooled analysis could not fully adjust for this heterogeneity, underscoring the need for vigilant lifelong imaging surveillance and individualized surgical thresholds in LDS.

## Introduction

Marfan syndrome (MFS) and Loeys–Dietz syndrome (LDS) are connective tissue disorders that present multiple similarities, including autosomal dominance, pathogenic variants in specific genes, high risk of vascular aneurysm and aortic dissection, and notably the manifestation of systemic features. 1​

Long-term survival in patients with MFS and LDS greatly depends on cardiovascular complications, particularly the development of proximal or distal aortic aneurysms or dissections, as well as the need for mitral valve repair or subsequent reoperations. 2​ 3​ ​4​ For that reason, surgical intervention has become the standard management for both conditions to avoid aortic dissection and has improved survival. 5​ ​6​

Controversy persists regarding the postoperative progression of MFS and LDS patients following aortic surgery. Early research portrayed LDS as a more aggressive form of MFS, with dissections reported at younger ages and smaller aortic diameters. 7​ ​8​ Recent studies have revealed a more complex landscape in identifying milder LDS phenotypes with a slower disease course. 3​ ​9​ Total prophylactic aortic root replacement has proven effective in both syndromes. Emerging data suggest that patients with LDS may be more likely to require further surgical interventions, especially involving the aortic arch. 8​ However, LDS encompasses a spectrum of phenotypic severity influenced by underlying genotypes, which may result in variable clinical presentations and disease progression.

Despite these observations, no study has meta-analytically compared postoperative outcomes between MFS and LDS using reconstructed time-to-event data. Therefore, we performed a systematic review and meta-analysis evaluating long-term outcomes following aortic surgery in MFS and LDS patients, acknowledging that the pooled comparisons are unadjusted for individual-level covariates.

## Materials and Methods

This meta-analysis was registered with the National Institute for Health Research International Registry of Systematic Reviews (PROSPERO, CRD42024596096). Ethical approval of this quantitative synthesis was not required as no human or animal subjects were involved.

### Search Strategy

We performed a comprehensive literature search to identify studies reporting survival, freedom from reoperation, and freedom from dissection after aortic surgery in patients with MFS compared with LDS. Searches were run on September 9, 2024, in Ovid MEDLINE, Scopus, Web of Science, and the Cochrane Library, using equivalent Boolean logic adapted to each database’s native syntax. Searches were restricted to English-language publications, with no additional filters applied. Reference lists of all full-text-reviewed studies were manually screened to identify additional eligible studies. The complete search strategy is provided in **Supplementary Table S1** (available in the online version only).

### Study Selection and Data Extraction

The study selection followed the Preferred Reporting Items for Systematic Reviews and Meta-Analyses strategy (PRISMA). After deduplication, records were screened by two independent reviewers (S. W. and A. A.). Any discrepancies and disagreements were resolved by a third author (J. F.). Titles and abstracts were reviewed against predefined inclusion and exclusion criteria. Studies were considered for inclusion if they were written in English, reported long-term survival, and the data were displayed with a Kaplan–Meier curve showing direct comparison between patients with MFS compared with LDS after aortic surgery. Animal studies, abstracts, case reports, commentaries, editorials, expert opinions, conference presentations, and studies not reporting the outcomes of interest were excluded. The full texts were pulled for the selected studies for a second round of eligibility screening. References for articles selected were also reviewed for relevant studies not captured by the original search. Two reviewers (S. W. and A. A.) independently performed data extraction. Accuracy was verified by a third author (J. F.). The extracted variables included study characteristics (publication year, country, sample size, study design, follow-up, and the population adjustment modality; Table 1 ) as well as the demographic data (age, sex, initial aortic dissection, hypertension, previous history of smoking and bicuspid aortic valve [BAV] rate; **Supplementary Table S2** [available in the online version only]) and initial surgical strategies ( **Supplementary Table S3** [available in the online version only]). The included studies used several statistical adjustment methods to reduce confounding. These included case-matched comparisons, multivariable Cox regression models adjusting for factors such as age and baseline dissection and competing risk models using Fine and Gray’s subhazard regression or Gray’s test to account for reoperation and dissection events. Kaplan–Meier estimates with log-rank testing were commonly used to compare survival and event-free outcomes and one study applied receiver operating characteristic analysis to identify prognostic thresholds for key variables.

**Table:** **Table 1** Summary of included studies

Author	Year of publication	Country	Number of patients	Study design	Mean follow-up	Population comparability	Reported outcomes
Schoenhoff et al. 12​	2014	Switzerland	66	Retrospective, monocentric study	12.7±7 y	Not adjusted	Long-term survival Reoperation
Mühlstädt et al. 13​	2019	Germany	166	Retrospective, multicentric study	NR	Cox proportional hazard regression	Long-term survival Reoperation
Schoenhoff et al. 16​	2020	United States	335	Retrospective, multicentric study	6.7±5.4 y (MFS) vs. 6.2±5.4 y (LDS)	Cox proportional hazard regression	Long-term survival Dissection Reoperation
Seike et al. 14​	2020	Japan	123	Retrospective, monocentric study	127±76 mo (MFS) vs. 144±101 mo (LDS)	Cox proportional hazard regression	Long-term survival Dissection Reoperation
Robertson et al. 15​	2022	Australia	315	Retrospective, monocentric study	20.4±11.4 y (MFS) vs. 15.0±11.3 y (LDS)	Not adjusted	Long-term survival Dissection

Abbreviations: LDS, Loeys–Dietz syndrome; MFS, Marfan syndrome; NR, not reported.

Note: References are reported in the Supplementary Material (available in the online version only).

### Risk-of-bias Assessment

The revised Newcastle Ottawa Scale was systematically used to assess the included studies for risk of bias ( **Supplementary Table S4** [available in the online version only]). Two independent reviewers (A. Al. and J. F.) assessed risk of bias. When there was a disagreement, a third reviewer (T. C.) checked the data and made the final decision.

**Table:** **Table 2** Outcomes summary

Outcome	Number of studies	Number of patients	Effect estimate (95% CI, *p* -value)
Long-term survival based on the patient’s age	3	808	HR: 0.919; 95% CI: 0.58–1.45; *p* =0.717
Long-term survival based on the patient’s postoperative follow-up	3	493	HR: 0.997; 95% CI: 0.45–2.19; *p* =0.995
Freedom from dissection	3	765	HR: 0.331; 95% CI: 0.21–0.53; *p* <0.005
Freedom from reoperation	4	575	HR: 0.603; 95% CI: 0.40–0.90; *p* =0.014

Abbreviations: CI, confidence interval; HR, hazard ratio.

### Outcomes

The main outcome was long-term survival, which was evaluated according to both the patient’s age at the time of the outcome and the duration of follow-up. Additional outcomes were freedom from aortic dissection and reoperation after aortic surgery.

### Statistical Analysis

Statistical analyses were restricted to outcomes reported in at least three studies. We used the methods described by Guyot et al. 10​ and Wei and Royston 11​ to reconstruct individual patient data (IPD) from the Kaplan–Meier curves of all eligible studies for the long-term survival. Hazard ratios (HRs) reconstructed from the Kaplan–Meier curves represent unadjusted survival comparisons, as individual covariate data were not available. Although some included studies reported adjusted analyses using the Cox regression models, these could not be uniformly incorporated into the pooled analysis. Raster and Vector images of the Kaplan–Meier curves were preprocessed and digitized so that the values corresponding to specific time points with their corresponding survival information could be extracted. Where additional information (e.g., number-at-risk tables or total number of events) was available, they were used to further calibrate the accuracy of the time to events. 10​ ​11​ To confirm the quality of the timing of failure events captured, we thoroughly checked the consistency with the reported outcome data provided in the original publications. The Kaplan–Meier method was used to calculate the overall survival rate. The Cox proportional hazards regression model was used to assess the between-group differences. For these Cox models, the proportional hazards assumption was verified by plotting scaled Schoenfeld residuals, log−log survival plots and predicted versus observed survival functions. Survival curves were plotted using the Kaplan–Meier product-limit method and each group’s HRs and 95% confidence intervals (CIs) were calculated. Digitization was performed using WebPlotDigitizer (version 4.6; Ankit Rohatgi), and accuracy was cross-checked against the number-at-risk tables and total reported event counts in the original publications. MFS was used as the reference category in all Cox models; HRs below 1.0 for the freedom-from-dissection and freedom-from-reoperation endpoints therefore indicate a lower probability of remaining event-free (i.e., a higher hazard of dissection or reoperation) in the LDS group relative to MFS. Time zero was defined as the date of the index (initial) aortic operation reported in each cohort; postoperative dissection and reoperation events were pooled across proximal and distal locations and included endovascular reinterventions were reported by the original studies, consistent with the outcome definitions used in the primary publications.

To assess the robustness of the findings, a two-stage meta-analysis was performed using the reconstructed individual HRs from each study, providing a pooled estimate of the overall effect while accounting for between-study variability. Additionally, a sensitivity analysis was conducted using a Cox proportional hazards model with robust standard errors clustered by study to further adjust for potential within-study correlation. All statistical analyses were performed using R and STATA IC17.0 (StataCorp LLC).

## Results

### Study Characteristics

A total of 1,794 studies were retrieved from the systematic search, of which 5 met the criteria for inclusion in the final analysis, encompassing 1,005 patients (MFS: 778; LDS: 227). Fig. 1 shows the Preferred Reporting Items for Systematic Reviews and Meta-Analyses (PRISMA) flowchart for study selection. The included studies were published between 2014 and 2022. Two were multicentric and three were monocentric.

**Fig. 1:**
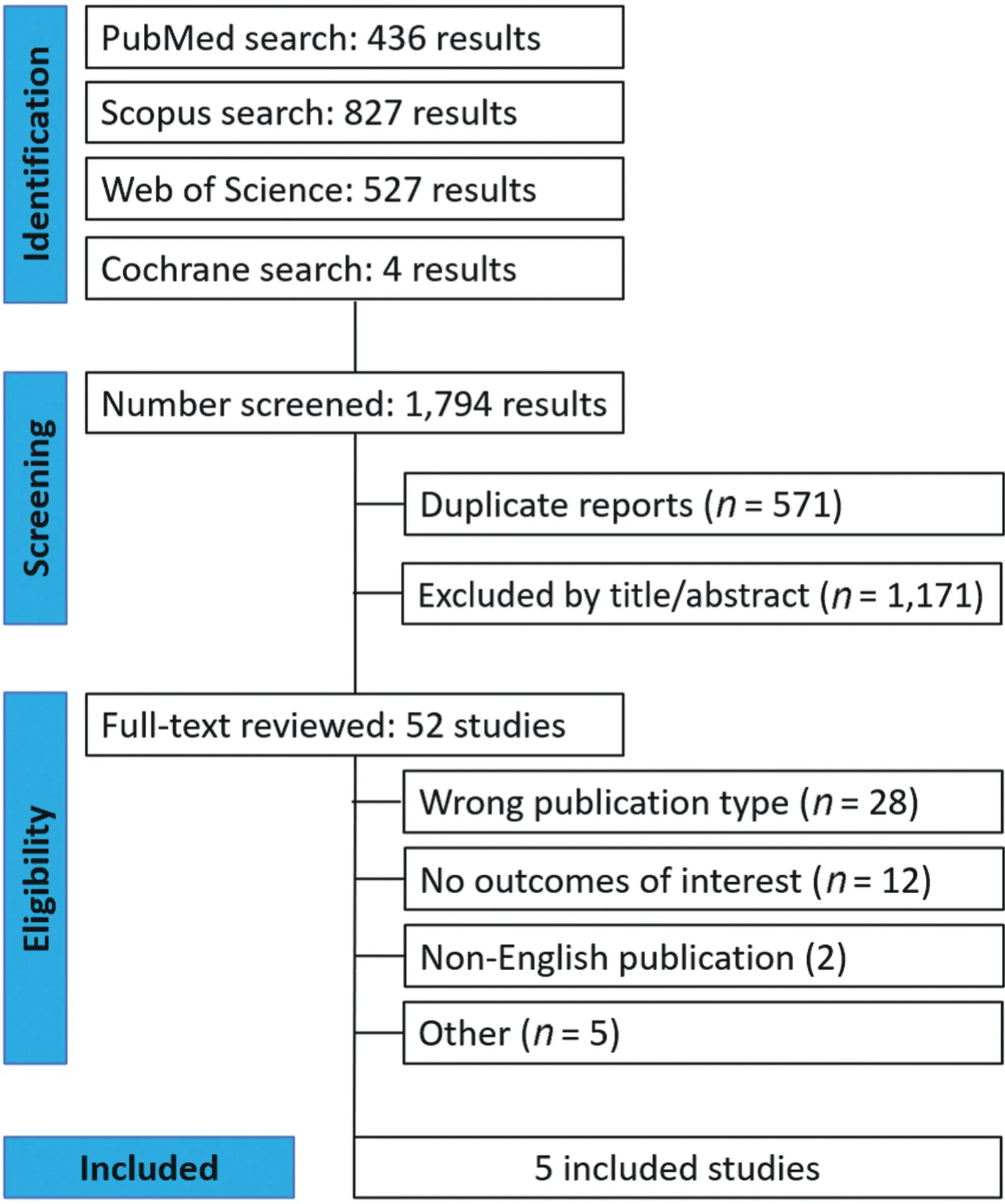
Preferred Reporting Items for Systematic Reviews and Meta-Analyses (PRISMA) flow diagram.

### Patient Characteristics

Table 1 summarizes the included studies, with sample sizes ranging from 68 to 335 patients. Detailed demographic and baseline characteristics are presented in **Supplementary Table S2** (available in the online version only). Across studies, the mean age of patients with MFS ranged from 22.1 to 40 years, whereas for LDS it ranged from 23 to 38 years. The proportion of female patients varied between 41.7 and 57.6% in MFS and 45.8 to 50% in LDS. The prevalence of initial aortic dissection at presentation ranged from 7.4 to 41.5% in MFS and 11 to 58.3% in LDS, whereas hypertension was reported in 0.35 to 28.3% of MFS and 0 to 33.3% of LDS cases. Smoking history was infrequently reported but reached 42% in MFS and 44% in LDS in the Schoenhoff et al. 2014 cohort. 12​ The proportion of BAV was low across studies, ranging from 0 to 8.1% in MFS and 0 to 34.4% in LDS, with some studies reporting none.

### Main Outcome

Table 2 summarizes the main findings of this analysis.﻿ Fig. 2A shows the survival databased on the patient’s age, where patient age at the time of the event is used as the time axis, allowing comparison of age-specific mortality between groups independent of differences in follow-up duration. There was no significant difference between MFS and LDS groups (HR: 0.919; 95% CI: 0.58–1.45; *p* =0.717). Fig. 2B presents the survival databased on postoperative follow-up. The outcome was similar in both groups (HR: 0.997; 95% CI: 0.45–2.19; *p* =0.995).

**Fig. 2:**
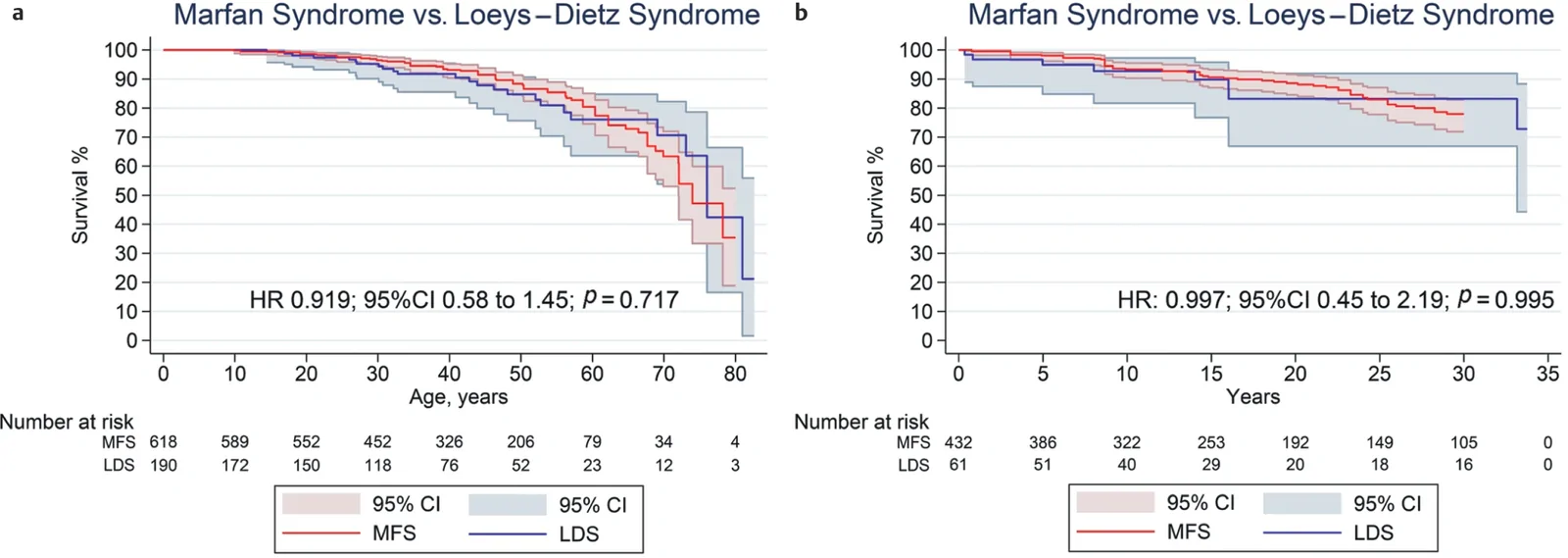
Pooled Kaplan–Meier curves showing MFS and LDS in the long-term survival based on ( **a** ) the patient’s age and based on ( **b** ) the patient’s postoperative follow-up. CI, confidence interval; HR, hazard ratio; LDS, Loeys–Dietz syndrome; MFS, Marfan syndrome.

### Sensitivity Analysis

To address the robustness of our findings, a leave-one-out analysis ( **Supplementary Fig. S1** [available in the online version only]) was performed for the primary outcome. Excluding any single study did not materially change the overall direction of the results. However, when the study by Robertson et al. was omitted, the association reached statistical significance (HR: 0.51, 95% CI: 0.27–0.97; *p* =0.038), suggesting that this study contributed to some variability in the pooled estimate. The same tendency was detected when addressing the individual HR of the studies in the two-stage meta-analysis using random-effects model ( **Supplementary Fig. S2** [available in the online version only]). The Schoenfeld residuals test did not indicate a significant violation of the proportional hazards assumption ( **Supplementary Fig. S3** [available in the online version only]).

In the shared-frailty Cox model, which accounted for study-level heterogeneity, the HR for long-term survival comparing MFS and LDS was 0.92 (95% CI: 0.48–1.77; *p* =0.80) when stratified by patient age and 0.99 (95% CI: 0.56–1.77; *p* =0.99) when stratified by follow-up duration. Both models converged appropriately, and no evidence of significant between-study heterogeneity was detected.

### Additional Outcomes

Fig. 3 shows the data on freedom from dissection. The reconstructed time-to-event Kaplan–Meier curve shows that patients with MFS had a higher rate of freedom from dissection in comparison with patients with LDS (HR: 0.331; 95% CI: 0.21–0.53; *p* <0.005). Fig. 4 demonstrates the data on the freedom from reoperation. Patients with MFS demonstrated a higher rate of freedom from reoperation compared with patients with LDS (HR: 0.603; 95% CI: 0.40–0.90; *p* =0.014).

**Fig. 3:**
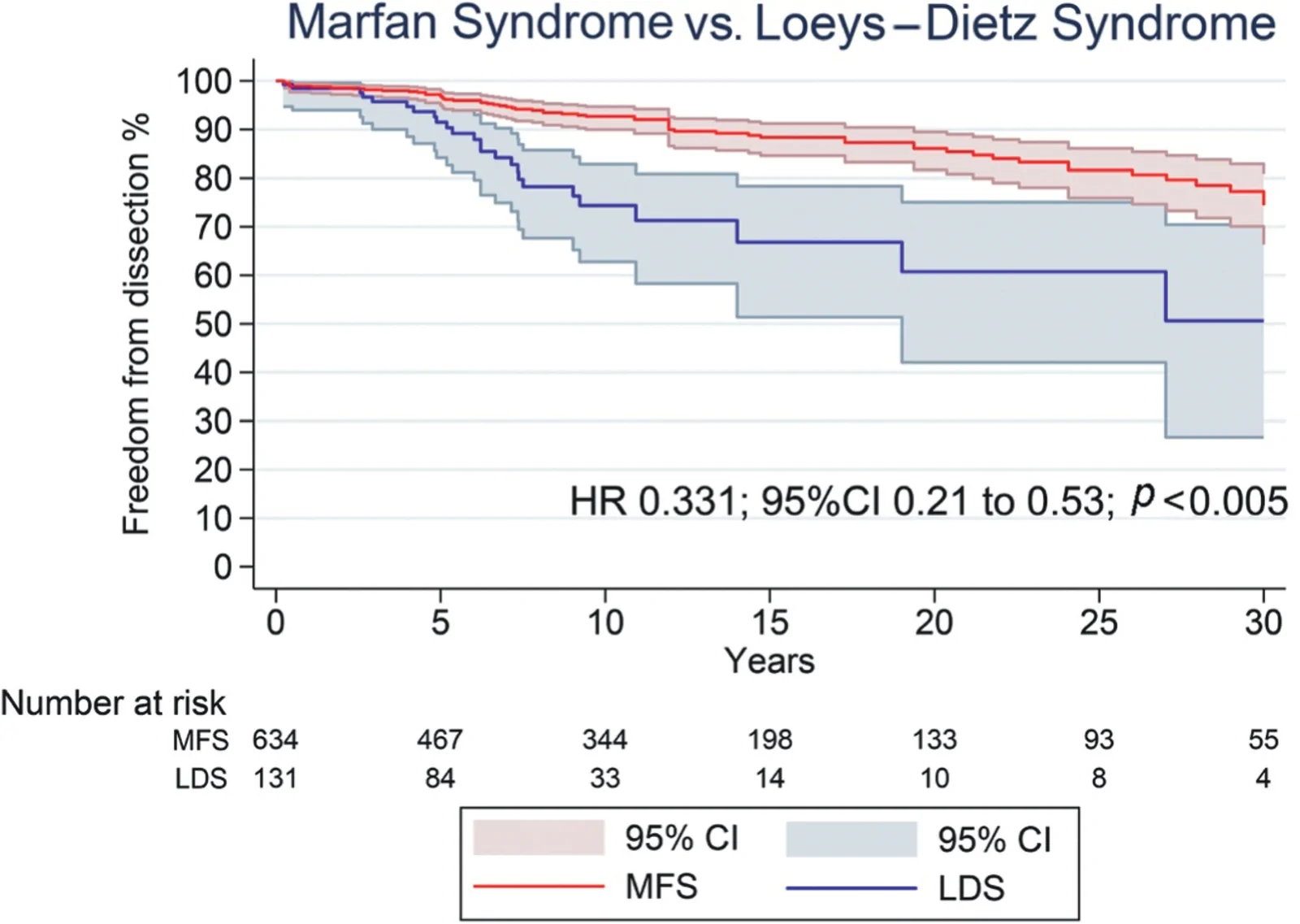
Pooled Kaplan–Meier curves for freedom from dissection. CI, confidence interval; HR, hazard ratio; LDS, Loeys–Dietz syndrome; MFS, Marfan syndrome.

**Fig. 4:**
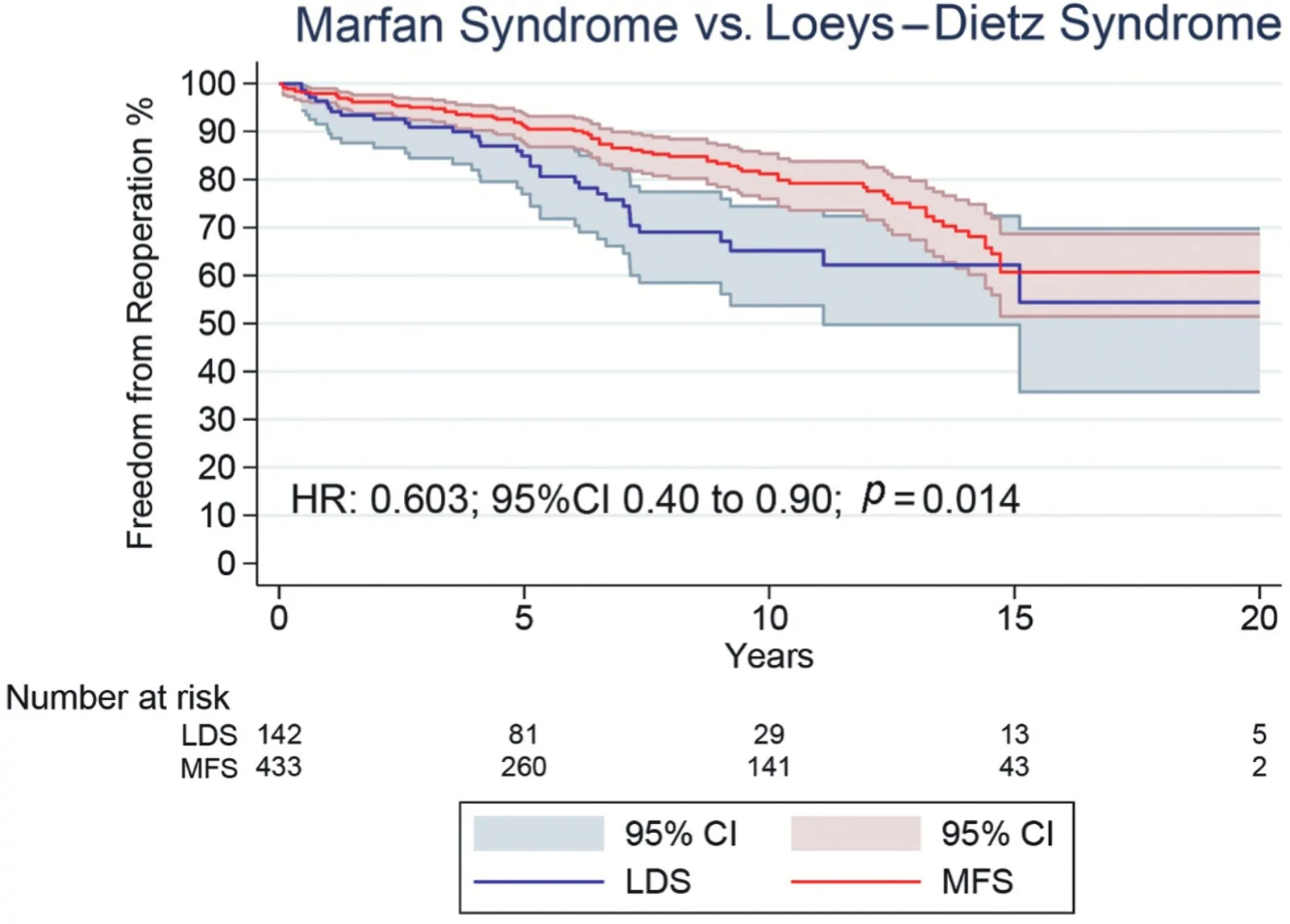
Pooled Kaplan–Meier curves for freedom from reoperation. CI, confidence interval; HR, hazard ratio; LDS, Loeys–Dietz syndrome; MFS, Marfan syndrome.

### Risk-of-bias Assessment

The risk of bias is summarized in **Supplementary Table S4** (available in the online version only). All included studies demonstrated overall good methodological quality. The majority achieved the maximum scores for Selection (3–4 stars) and Outcome/Exposure (3 stars), indicating appropriate cohort definition, outcome ascertainment, and follow-up adequacy. Comparability domains consistently received 2 stars across studies, reflecting adequate adjustment for key confounders such as age and baseline dissection status.

## Discussion

This work shows that, despite similar long-term survival, patients with LDS had a significantly higher risk of dissection and reoperation compared with those with MFS. These comparisons reflect unadjusted, reconstructed time-to-event data, as individual-level covariate information was not available for pooling. Death represents a competing risk for both dissection and reoperation; this is addressed further in the study limitations. These findings suggest that closer clinical monitoring may be warranted in LDS patients to improve long-term prognosis.

The survival rate in our findings, based on age and follow-up, is consistent with the included individual studies. Four out of five studies in our meta-analysis reported no significant differences between MFS and LDS after aortic surgery. 12​ 13​ 14​ ​15​ Nevertheless, one study observed significantly better long-term survival for the MFS group in the unadjusted analysis. Interestingly, after adjustment for age and prior/initial dissection, survival did not differ between the groups. The difference was primarily driven by the strong influence of aortic dissection on survival outcomes, rather than by the underlying syndrome itself. 16​

The proportion of patients presenting with aortic dissection likely influenced the overall outcomes of our analysis. As most patients in the included studies had MFS, the lower baseline prevalence of dissection compared with LDS may have contributed to the similar survival observed between groups, despite the higher dissection and reoperation rates in LDS. This imbalance underscores the importance of baseline disease severity in interpreting comparative outcomes between both syndromes.

Our results show that the freedom from dissection rate was significantly higher in the MFS group, indicating that more LDS patients experienced a dissection. Seike et al. similarly reported that the cumulative incidence of aortic dissection after initial aortic surgery was higher in the LDS group. The cumulative incidence of first aortic arch reoperation was higher in LDS, whereas the cumulative incidences of first aortic root and descending aorta reoperations were similar between groups. 14​ In contrast, Robertson et al. reported a significantly worse initial presentation of LDS patients, as aortic dissection was the first event in 34% of cases, compared with only 8% in MFS. However, more dissections occurred in the MFS group during further follow-up up to the age of 60 years. An explanation for these results is the observation that most follow-up dissections in the MFS group were type B and 19 occurred after a previous aortic root/ascending aorta repair. Additionally, prophylactic aortic surgery frequently occurred as a first event and survival among individuals enrolled in follow-up surveillance was comparable across aortopathies. 15​

Importantly, the freedom from reoperation was significantly higher in MFS compared with LDS in our study, indicating that more LDS patients required secondary interventions. Seike et al. 14​ and Schoenhoff et al. 16​ reported similar findings, with higher overall reoperation rates in LDS, mainly due to subsequent aortic arch procedures in patients without prior dissection. The indications for reoperation across the included studies primarily reflected progressive or residual aortic pathology rather than technical failure. Most reoperations were performed for distal aortic dilation, expansion of true or false lumens following dissection, or development of new thoracic aneurysms. In LDS, continuous enlargement of the distal ascending aorta and proximal arch, sometimes complicated by pseudoaneurysm formation or suture line dehiscence, was a common cause of reintervention. Elective aortic root or ascending repair was also performed at lower diameter thresholds in LDS, consistent with its more aggressive vascular phenotype. These findings underscore the need for long-term surveillance and individualized surgical timing, particularly in patients with LDS. Mitral valve surgery was occasionally required in both groups for severe regurgitation or, less frequently, for acute endocarditis.

The reasons behind the higher dissection and reoperation rates in LDS despite comparable survival likely reflect intrinsic disease aggressiveness, differences in pathophysiology, and the timing and extent of surgical interventions. LDS is characterized by more diffuse and aggressive arterial involvement, with aneurysms and dissections occurring at smaller diameters and at younger ages than in MFS. 14​ The implementation of earlier and more extensive surgical strategies in LDS, particularly prophylactic or aggressive arch repair, appears to mitigate the survival disadvantage that would otherwise result from this phenotype. 3​ These findings suggest that vigilant postoperative surveillance likely contributes to maintaining comparable survival outcomes between LDS and MFS, although the specific impact of earlier or more extensive interventions remains uncertain. 17​

Contemporary clinical management likely accounts for these observations. Enrolment in surveillance programs and the frequent use of prophylactic aortic surgery as a first event are consistent with the comparable survival rates observed between LDS and MFS. 15​ Furthermore, recommendations for aggressive arch surgery during the initial operation in LDS are consistent with the observation of comparable long-term survival after the initial repair. 14​

A heterogeneity factor in this meta-analysis was the genotype definition of LDS. Schoenhoff et al., 12​ Robertson et al., 15​ and Seike et al. 14​ strictly limited the LDS analysis to TGFBR1/2 and excluded SMAD3, thereby illustrating a more aggressive LDS profile. Mühlstädt et al. 13​ and Schoenhoff et al. 16​ included TGFBR1/2 and SMAD3 in the LDS spectrum. Therefore, depending on the genotype mix, aortic dissection and reoperation rates may shift.

A pooled subgroup analysis stratified by LDS genotype was not performed, as individual-level genotype data were not consistently available across studies. The pattern described above is therefore hypothesis-generating rather than a formally tested subgroup effect. Although long-term survival rates are similar, LDS has a higher risk of dissections and reoperations. These findings underline the importance of careful long-term monitoring and raise the hypothesis that earlier or more extensive arch strategies might reduce event rates in LDS, a question that should be addressed in future studies.

### Study Limitations

This meta-analysis is the first to reconstruct time-to-event data to examine outcomes after aortic surgery in patients with connective tissue disorders. However, several limitations must be acknowledged. First, a heterogeneous definition of LDS genotype across studies carries a risk of misclassification and may have led to variations in the reported rates of aortic dissections and reoperations. The intrinsic heterogeneity within the LDS population further compounds this issue, as different genotypes are associated with variable phenotypic expression and degrees of vascular aggressiveness, which could not be fully captured in the pooled analysis and may have contributed to variability in the observed outcomes.

Most included cohorts were small, retrospective studies and only five met the inclusion criteria, resulting in a modest overall sample size and limited statistical power. Consequently, wide CIs indicate uncertainty around some estimates, which should be interpreted with caution. This small number of studies also precluded formal meta-regression, subgroup analyses, and reliable assessment of publication bias, as tests such as Egger’s or funnel plots have limited interpretability under these conditions.

Although the inclusion criteria mentioned risk-adjusted studies, the pooled estimates were primarily derived from reconstructed Kaplan–Meier databased on unadjusted survival curves, as adjusted HRs were inconsistently reported. Therefore, the HRs obtained reflect unadjusted comparisons between MFS and LDS, limiting causal inference. As our analysis was based on aggregated study-level data rather than individual patient-level data, the ability to adjust for confounders or explore study-level covariates was restricted. Differences in baseline characteristics, surgical indications and institutional practices may therefore have influenced the observed outcomes. Residual confounding cannot be fully excluded despite the use of random-effects models. Furthermore, the included cohorts comprised a mixture of patients who underwent prophylactic aortic surgery and those operated on after an initial dissection. Crucially, information on whether prophylactic root replacement had been performed was not uniformly available across studies. Prophylactic repair is known to reduce the subsequent risk of dissection. This heterogeneity in surgical indication may have influenced the freedom-from-dissection and freedom-from-reoperation estimates and could not be adjusted for in the pooled analysis.

In addition, the reconstruction of Kaplan–Meier curves without midterm numbers at risk may have introduced imprecision in the time-to-event estimates, particularly at longer follow-up periods, where few patients remained at risk. Death, reoperation, and aortic dissection act as competing risks that were inconsistently distinguished across the included studies, particularly regarding new versus chronic residual dissections. Consequently, the reconstructed freedom-from-event curves should be interpreted as descriptive estimates of event burden rather than fully adjusted risk models. Furthermore, detailed morphometric data, such as midterm aortic diameters or measurements of graft durability, were not consistently available, precluding additional analyses of postoperative aortic remodeling. Similarly, the lack of standardized reporting prevented differentiation between proximal and distal reoperations or between surgical and endovascular reinterventions, which would have provided a more refined understanding of long-term outcomes.

Finally, this study-level approach cannot capture patient-level heterogeneity, and the observational design of the included studies introduces inherent risks of selection and reporting bias. Future investigations using IPD meta-analysis are warranted to enable more detailed adjustment for clinical and surgical covariates, to test model assumptions and to provide more robust causal interpretation of the differences between LDS and MFS cohorts.

## Conclusion

This work showed that although long-term survival is similar between MFS and LDS patients, those with LDS face a significantly higher risk of aortic dissection and reoperation. This elevated risk may be partly explained by the intrinsic genotypic heterogeneity of the LDS population. However, our pooled analysis could not fully adjust for this heterogeneity, underscoring the need for vigilant lifelong imaging surveillance and individualized surgical thresholds in LDS.

## Data Availability

The data underlying this article are available in the article and in its online supplementary material. The reconstructed individual patient time-to-event datasets are available from the corresponding author upon reasonable request.

## Supplementary Material

Supplementary Material Zusatzmaterial

## References

[1] Pyeritz R E Heritable thoracic aortic disorders Curr Opin Cardiol 2014 29 01 97 102 https://pubmed.ncbi.nlm.nih.gov/24284977 10.1097/HCO.0000000000000023 24284977

[2] Engelfriet P M Boersma E Tijssen J G Bouma B J Mulder B J Beyond the root: dilatation of the distal aorta in Marfan’s syndrome Heart 2006 92 09 1238 1243 https://pubmed.ncbi.nlm.nih.gov/16488927 10.1136/hrt.2005.081638 PMC1861183 16488927

[3] Attias D Stheneur C Roy C Comparison of clinical presentations and outcomes between patients with TGFBR2 and FBN1 mutations in Marfan syndrome and related disorders Circulation 2009 120 25 2541 2549 https://pubmed.ncbi.nlm.nih.gov/19996017 10.1161/CIRCULATIONAHA.109.887042 19996017

[4] Iba Y Minatoya K Matsuda H Surgical experience with aggressive aortic pathologic process in Loeys-Dietz syndrome Ann Thorac Surg 2012 94 05 1413 1417 https://pubmed.ncbi.nlm.nih.gov/22921234 10.1016/j.athoracsur.2012.05.111 22921234

[5] Cameron D E Alejo D E Patel N D Aortic root replacement in 372 Marfan patients: evolution of operative repair over 30 years Ann Thorac Surg 2009 87 05 1344 1349 https://pubmed.ncbi.nlm.nih.gov/19379862 10.1016/j.athoracsur.2009.01.073 19379862

[6] Schoenhoff F S Jungi S Czerny M Acute aortic dissection determines the fate of initially untreated aortic segments in Marfan syndrome Circulation 2013 127 15 1569 1575 https://pubmed.ncbi.nlm.nih.gov/23493319 10.1161/CIRCULATIONAHA.113.001457 23493319

[7] Loeys B L Schwarze U Holm T Aneurysm syndromes caused by mutations in the TGF-beta receptor N Engl J Med 2006 355 08 788 798 https://pubmed.ncbi.nlm.nih.gov/16928994 10.1056/NEJMoa055695 16928994

[8] Williams J A Loeys B L Nwakanma L U Early surgical experience with Loeys-Dietz: a new syndrome of aggressive thoracic aortic aneurysm disease Ann Thorac Surg 2007 83 02 S757 S763, discussion S785–S790https://pubmed.ncbi.nlm.nih.gov/17257922 10.1016/j.athoracsur.2006.10.091 17257922

[9] Jondeau G Ropers J Regalado E International registry of patients carrying TGFBR1 or TGFBR2 mutations: results of the MAC (Montalcino Aortic Consortium) Circ Cardiovasc Genet 2016 9 06 548 558 https://pubmed.ncbi.nlm.nih.gov/27879313 10.1161/CIRCGENETICS.116.001485 PMC5177493 27879313

[10] Guyot P Ades A E Ouwens M J Welton N J Enhanced secondary analysis of survival data: reconstructing the data from published Kaplan-Meier survival curves BMC Med Res Methodol 2012 12 9 https://pubmed.ncbi.nlm.nih.gov/22297116 10.1186/1471-2288-12-9 PMC3313891 22297116

[11] Wei Y Royston P Reconstructing time-to-event data from published Kaplan-Meier curves Stata J 2017 17 04 786 802 https://pubmed.ncbi.nlm.nih.gov/29398980 PMC5796634 29398980

[12] Schoenhoff F S Mueller C Czerny M Outcome of aortic surgery in patients with Loeys-Dietz syndrome primarily treated as having Marfan syndrome Eur J Cardiothorac Surg 2014 46 03 444 449, discussion 449https://pubmed.ncbi.nlm.nih.gov/24499874 10.1093/ejcts/ezu002 24499874

[13] Mühlstädt K De Backer J von Kodolitsch Y Case-matched comparison of cardiovascular outcome in Loeys-Dietz syndrome versus Marfan syndrome J Clin Med 2019 8 12 8 https://pubmed.ncbi.nlm.nih.gov/31795342 10.3390/jcm8122079 PMC6947024 31795342

[14] Seike Y Matsuda H Inoue Y The differences in surgical long-term outcomes between Marfan syndrome and Loeys-Dietz syndrome J Thorac Cardiovasc Surg 2022 164 01 16 2500 https://pubmed.ncbi.nlm.nih.gov/32891455 10.1016/j.jtcvs.2020.07.089 32891455

[15] Robertson E N Bannon P G Jeremy R W Long-term outcomes in heritable thoracic aortic disease Front Cardiovasc Med 2022 9 1009947 https://pubmed.ncbi.nlm.nih.gov/36312254 10.3389/fcvm.2022.1009947 PMC9606219 36312254

[16] Schoenhoff F S Alejo D E Black J H Management of the aortic arch in patients with Loeys-Dietz syndrome J Thorac Cardiovasc Surg 2020 160 05 1166 1175 https://pubmed.ncbi.nlm.nih.gov/31627951 10.1016/j.jtcvs.2019.07.130 31627951

[17] Kim H Lee J H Chung S R Impact of early diagnosis on surgical outcomes in patients with Loeys-Dietz syndrome Front Cardiovasc Med 2024 11 1429222 https://pubmed.ncbi.nlm.nih.gov/39221421 10.3389/fcvm.2024.1429222 PMC11363540 39221421

