# Supplementary material for: Marfan Syndrome versus Loeys–Dietz Syndrome after Aortic Surgery: A Systematic Review and Meta-analysis of Reconstructed Time-to-Event Data
Source: Aorta (Stamford). 2026 Oct 8;14:a29656400. doi: 10.1055/a-2965-6400 (PMC13657142; doi:10.1055/a-2965-6400)

Supplementary Table 1. Complete Search strategy for each database

Ovid MEDLINE

Search: **("marfan"[All Fields] OR "marfan s"[All Fields] OR "marfans"[All Fields]) AND "Loeys-Dietz"[All Fields]**

("marfan"[All Fields] OR "marfan s"[All Fields] OR "marfans"[All Fields]) AND "Loeys-Dietz"[All Fields]

Scopus

TITLE-ABS-KEY ((marfan OR "marfan's" OR marfans OR marfan*) AND ("Loeys-Dietz" OR "Loeys Dietz"))

Web of Science

TS=((marfan OR "marfan's" OR marfans OR marfan*) AND ("Loeys-Dietz" OR "Loeys Dietz"))

Cochrane Library

(marfan OR "marfan's" OR marfans OR marfan*) AND ("Loeys-Dietz" OR "Loeys Dietz")

Supplementary Table 2. Demographics of included patients from the selected studies.

Study	Age (mean±SD)		Female (%)		Initial Aortic dissection (%)		Hypertension (%)		Smoker (%)		Bicuspid Aortic Valve (%)	
	**MFS**	**LDS**	**MFS**	**LDS**	**MFS**	**LDS**	**MFS**	**LDS**	**MFS**	**LDS**	**MFS**	**LDS**
Schoenhof, 2014 (1)	35 ± 15	34 ± 13	NR	NR	37	33	NR	NR	42	44	NR	NR
Mühlstadt, 2019 (2)	40 ± 18	38 ± 18	48	48	13.3	13.3	NR	NR	NR	NR	0	6
Schoenhof, 2020 (3)	31 ± 16	23 ± 20	NR	NR	7.4	11	NR	NR	7.4	11	NR	NR
Seike, 2022 (4)	33 ± 7.5	28 ± 11	57.6	45.8	41.5	58.3	28.3	33.3	41.4	58.3	0	0
Robertson, 2022 (5)	22.1 ± 16.6	30.7 ± 22.7	41.7	50	8	34	0.35	0	8.1	34.4	NR	NR

Supplementary Table 3. Initial Surgical Strategies for Aortic Disease (LDS vs MFS)

Study (Year)	Surgical Strategy	LDS	MFS
Seike (2022)	Total Root Repair (Bentall + VSRR)	12 (50.0%)	85 (85.9%)
	Valve-Sparing Root Replacement (VSRR)	10 (41.7%)	57 (57.7%)
	Bentall Procedure (Substitution)	2 (8.3%)	28 (28.3%)
	Ascending Replacement + HAR (Hemiarch)	1 (4.2%)	0
	Ascending Replacement + TAR (Total Arch)	2 (8.4%)	4 (4.0%)
	Distal/Thoracoabdominal Replacement	9 (37.5%) (TAAAR: 3 (12.5%); Descending: 6 (25.0%))	10 (10.1%) (TAAAR: 1 (0.9%); Descending: 6 (6.1%))
Schoenhoff (2019)	Aortic Root Replacement (Total)	96%	99%
	Valve-Sparing Surgery (VSRR)	71%	63%
	1º Arch Repair (during Elective Root Surgery)	5%	0.40%
	1º Arch Repair (during Dissection Surgery)	8%	42%
Mühlstädt (2019)	Proximal Aortic Surgery (Total)	33 (40%)	37 (45%)
	Aortic Root Reconstruction (VSRR)	23 (70%)	19 (51%)
	Aortic Root Replacement (Mechanical Valve)	7 (21%)	15 (41%)
	Surgery Involving Arch (Extension)	2 (6%)	5 (14%)
Schoenhoff (2013)	Valve-Sparing Procedures (VSRR)	11%	23%
	Modified Bentall Procedures	Trend: 78%	Trend: 47%
	Simultaneous Total Arch Replacement (AAD)	None (0%)	12%
	Isolated Ascending Aorta Replacement (Early cases)	11%	14%

AAD: Acute Aortic Dissection; HAR: Hemiarch Replacement; TAAAR: Thoracoabdominal Aortic Repair; TAR: Total Aortic Arch Replacement; VSRR: Valve-Sparing Root Replacement.

Supplementary Table 4. Assessment of risk of bias using the Newcastle Ottawa Scale.

STUDY	SELECTION	COMPARABILITY	OUTCOME/ EXPOSURE
Schoenhoff, 2014	***	**	***
Mühlstadt, 2019	****	**	***
Schoenhoff, 2020	****	**	***
Seike, 2022	****	**	***
Robertson, 2022	****	**	***

LDS = Loeys-Dietz syndrome; MFS = Marfan syndrome; NR = Not reported.

Supplementary Figure 1. Leave one out analysis for primary outcome. A) Overall survival – Age; B) Overall survival – Follow-up

A

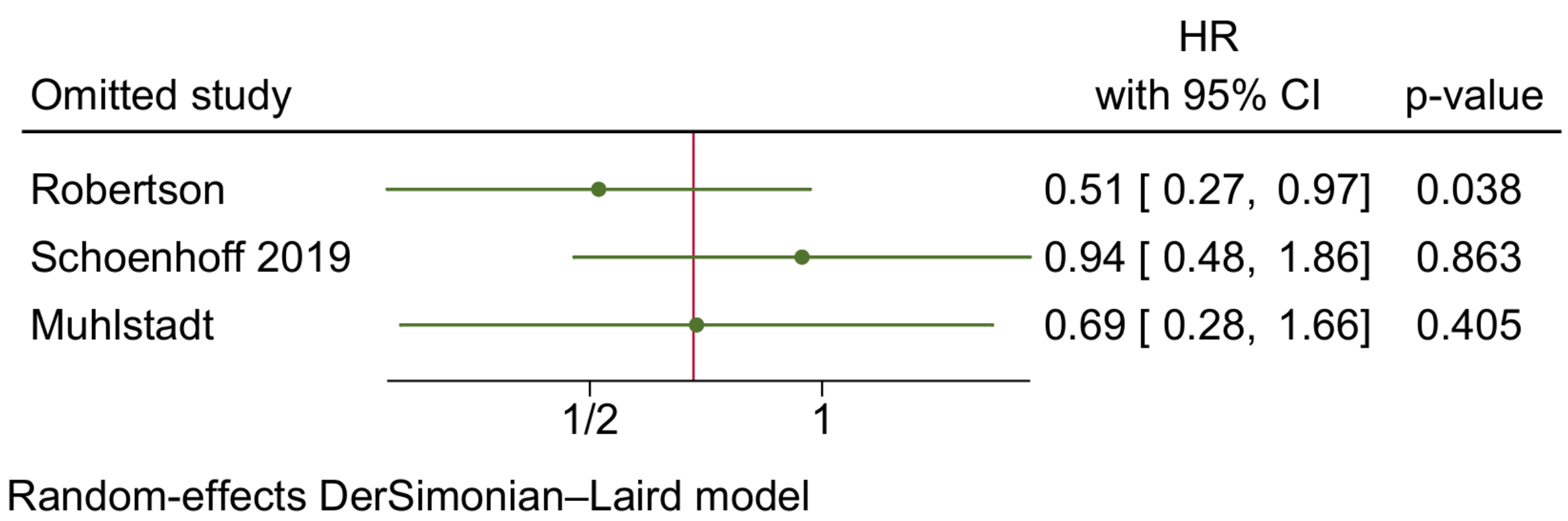

B

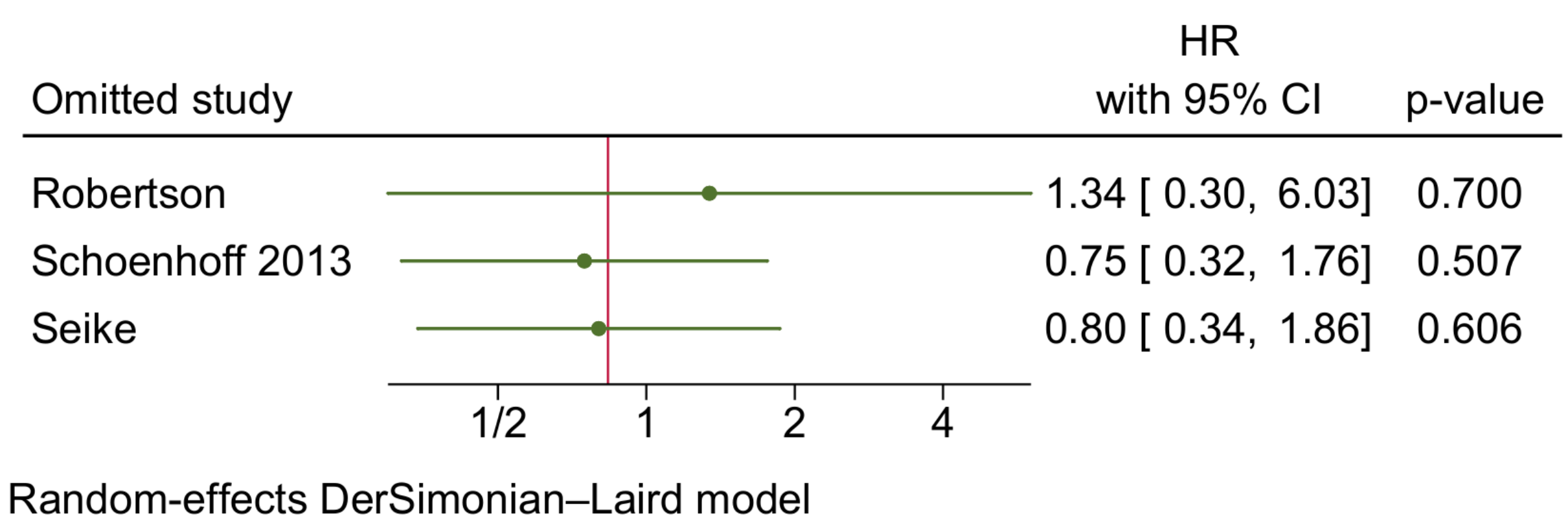

Supplementary Figure 2. Two stage meta-analyses for primary outcome. A) Overall survival – Age; B) Overall survival – Follow-up

A

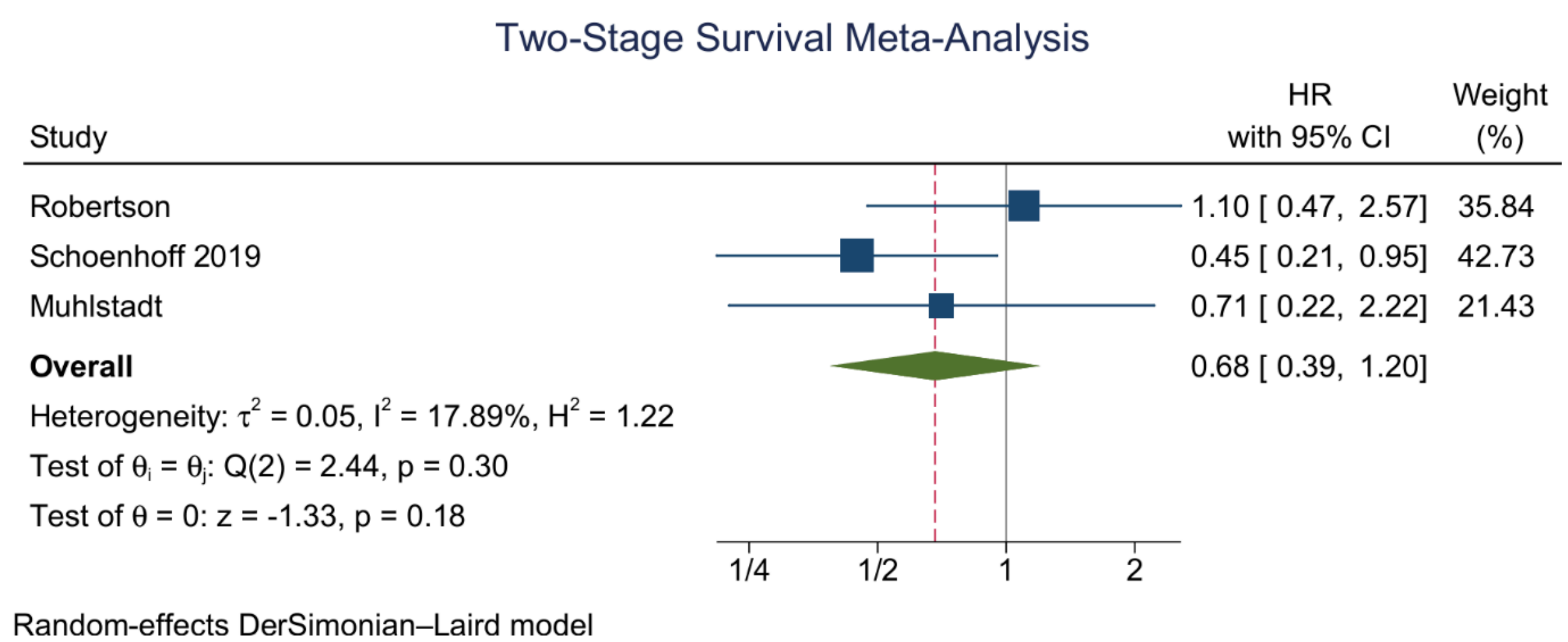

B

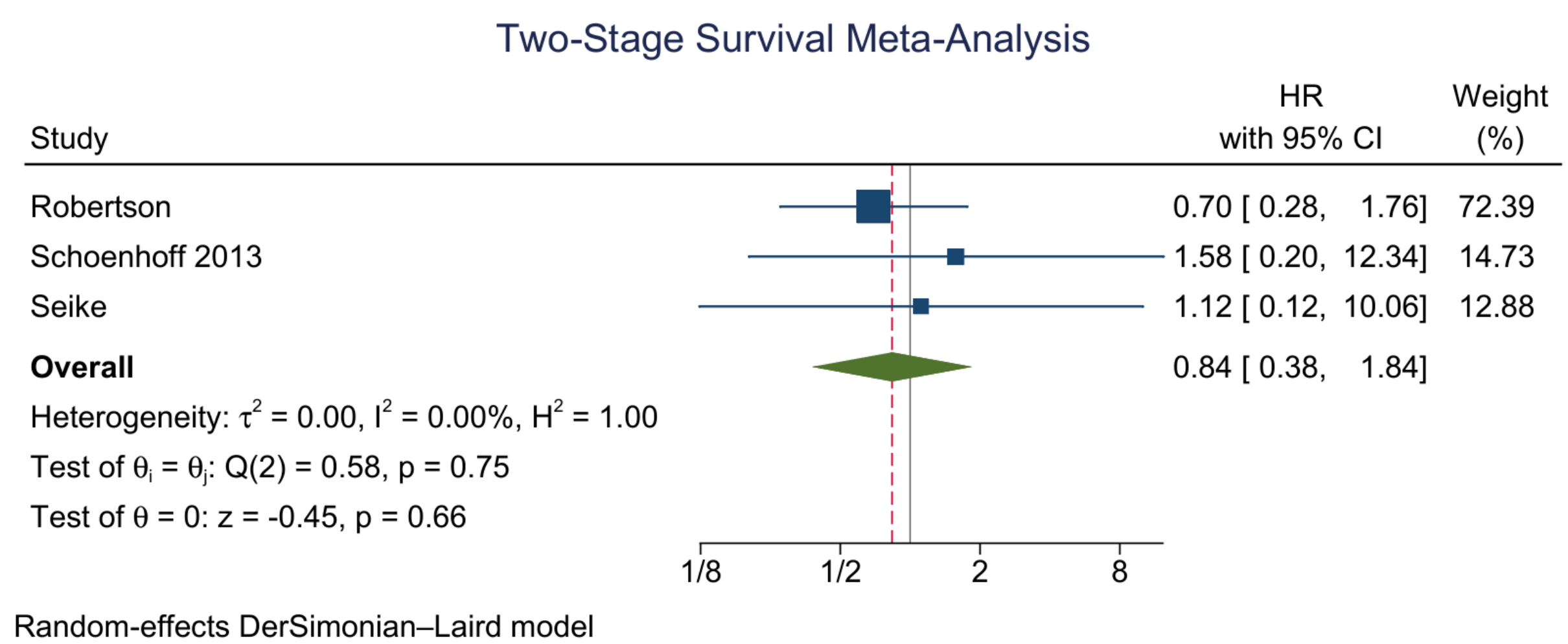

Supplementary Figure 3. Proportional hazards assumption assessment using Schoenfeld residuals for primary outcome A) Overall survival – Age; B) Overall survival – Follow-up

A

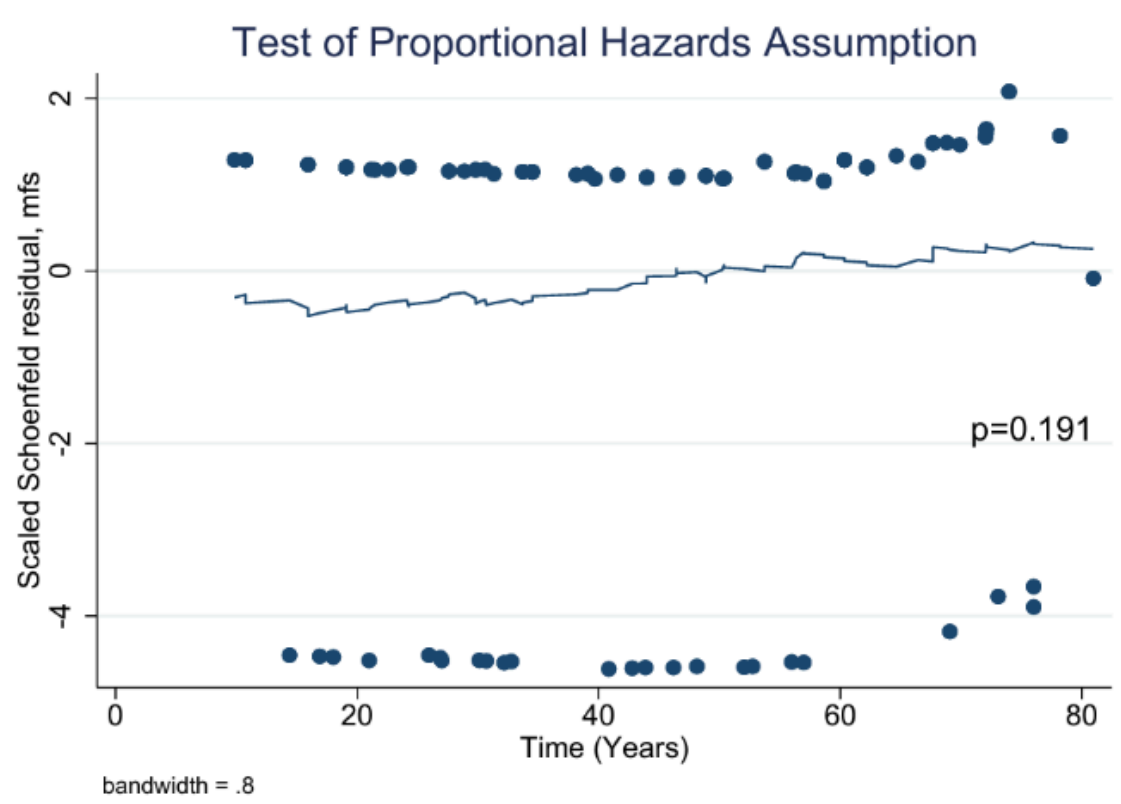

B

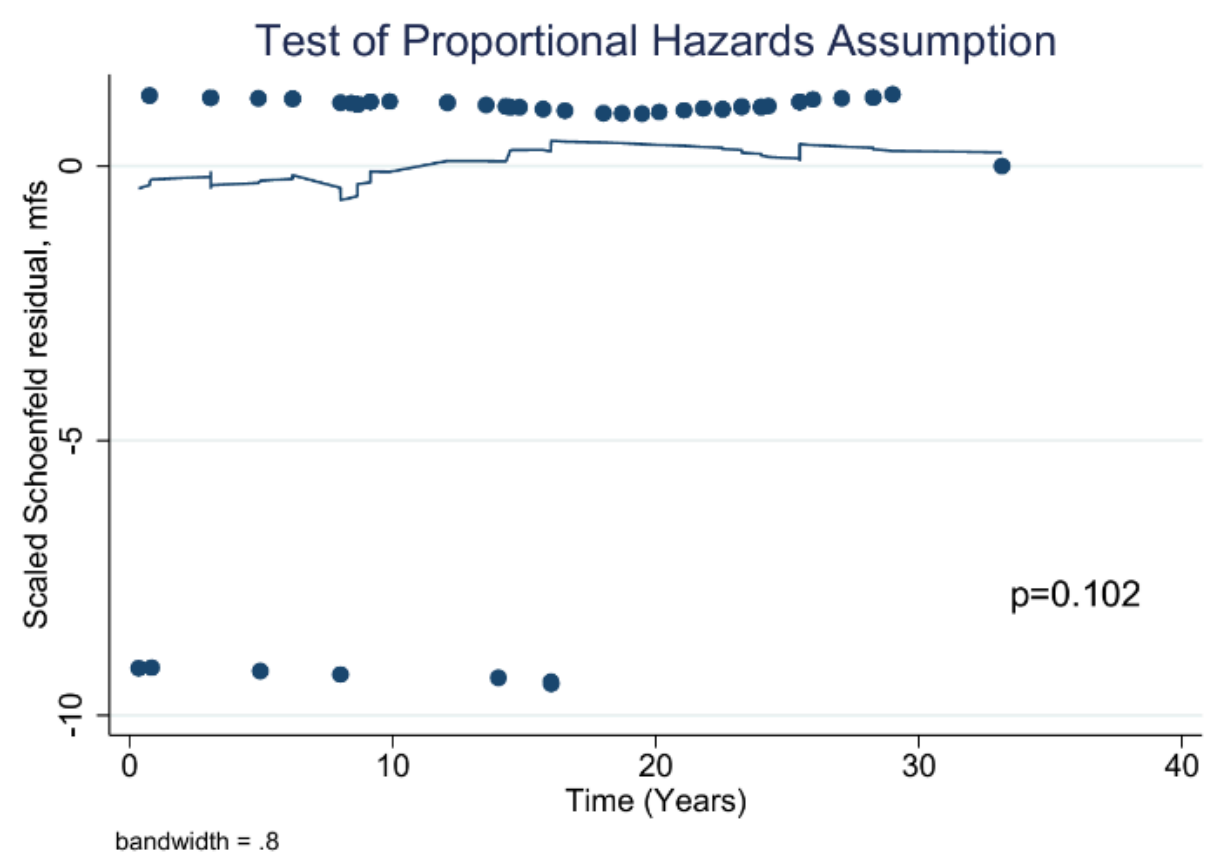

Supplement: Supplementary file 1 [file 10-1055-a-2965-6400_29706002.pdf]
